# Impact of home healthcare reform on place of death for people with dementia: A nationwide cohort study accounting for cultural factors of impending death discharge

**DOI:** 10.1017/S1478951526102491

**Published:** 2026-05-11

**Authors:** Chia-Hung Chen, Elizabeth L. Sampson, Jung-Yu Liao, Chung-Han Ho, Wei-Zhe Tseng, Irene Petersen, Yi-Chi Wang, Yi-Chen Lai, Yu-Han Chen, Hung-Yi Chiou, Chao A. Hsiung, Sang-Ju Yu, Christine Ritchie, Ping-Jen Chen

**Affiliations:** 1 Department of Family Medicine, Far Eastern Memorial Hospital, New Taipei City, Taiwan; 2 Master program of Transdisciplinary Long Term Care, School of Medicine, Fu Jen Catholic University, New Taipei City, Taiwan; 3 Academic Centre for Healthy Ageing, Whipps Cross Hospital, Barts Health NHS Trust, London, UK; 4 Centre for Psychiatry and Mental Health, Wolfson Institute of Population Health, Queen Mary University of London, London, UK; 5 Department of Liaison Psychiatry, Royal London Hospital, East London Foundation Trust, London, UK; 6 Department of Health Promotion and Health Education, National Taiwan Normal University, Taipei, Taiwan; 7 Department of Medical Research, Chi-Mei Medical Center, Tainan, Taiwan; 8 Department of Information Management, Southern Taiwan University of Science and Technology, Tainan, Taiwan; 9 Department of Family Medicine and Division of Geriatrics and Gerontology, Kaohsiung Medical University Hospital, Kaohsiung Medical University, Kaohsiung, Taiwan; 10 UCL Department of Primary Care and Population Sciences, University College London, London, UK; 11 Department of Emergency Medicine, An Nan Hospital, China Medical University, Tainan, Taiwan; 12 Department of Family Medicine, An Nan Hospital, China Medical University, Tainan, Taiwan; 13 Institute of Population Health Sciences, National Health Research Institutes, Zhunan, Miaoli County, Taiwan; 14 Taiwan Society of Home Health Care, Taipei, Taiwan; 15 Home Clinic Dulan, Taitung, Taiwan; 16 Mongan Institute Center for Aging and Serious Illness and the Division of Palliative Care and Geriatric Medicine, Massachusetts General Hospital and Harvard Medical School, Boston, Massachusetts, USA; 17 School of Medicine, College of Medicine, Kaohsiung Medical University, Kaohsiung, Taiwan; 18 National Center for Geriatrics and Welfare Research, National Health Research Institutes, Miaoli, Taiwan; 19 School of Medicine, College of Medicine, National Sun Yat-sen University, Kaohsiung, Taiwan

**Keywords:** People with dementia, place of death, home healthcare, Integrated Home-Based Medical Care, impending death discharge

## Abstract

**Background:**

Although home is frequently the preferred place of death, little is known regarding how home healthcare (HHC) influences this outcome for people with dementia (PWD), particularly within Asian contexts. This study investigates the impact of HHC and its 2016 “Integrated Home-Based Medical Care” reform on home death in Taiwan, explicitly accounting for the cultural phenomenon of “impending death discharge.”

**Methods:**

This nationwide retrospective cohort study utilized Taiwan’s National Health Insurance Research Database. We identified PWD decedents from 2011 to 2022 and conducted a nested case–control analysis. Cases (home deaths) and controls (hospital deaths) were matched 1:1 using propensity scores to balance demographics and health status. HHC models included pre-2016 primary HHC, post-2016 primary HHC, and the reformed “HBPC Plus” (Home-Based Primary Care Plus). The outcome was adjusted to include patients discharged terminally to die at home, reflecting distinctive cultural practices.

**Results:**

Among 209,468 decedents, 95,594 were selected after matching. Overall, HHC use was associated with higher odds of home death (adjusted odds ratio [aOR]: 1.17; 95% CI: 1.13–1.21). The reformed HBPC Plus program showed the strongest association compared to pre-2016 primary HHC (aOR: 1.63; 95% CI: 1.34–1.98). Crucially, this association strengthened further when accounting for impending death discharge (aOR: 1.82; 95% CI: 1.40–2.35). Higher visit frequency and services from hospital-based teams were also significantly linked to home death.

**Conclusions:**

HHC significantly increases the likelihood of home death among PWD in culturally influenced contexts. The 2016 reform, particularly the HBPC Plus program, proved highly effective. Policy components like flexible visit frequencies and enhanced hospital–physician coordination appear vital for supporting end-of-life care at home, offering key insights for policy planning in aging societies.

## Introduction

Dementia represents a leading cause of disability and mortality among older adults, imposing a significant burden not only on individuals but also on their caregivers, families, and healthcare systems (World Health Organization 2017; Wimo et al. 2023). As cognitive function and physical performance decline, people with dementia (PWD) often become increasingly frail and homebound, necessitating substantial long-term care (Gale et al. 2018; Sm-Rahman et al. 2022). To address these needs, home healthcare (HHC) – which typically involves professional services provided by registered nurses and physicians – delivers essential medical care to vulnerable individuals in the community (Zimbroff et al., 2021). HHC has been shown to be particularly beneficial for PWD, supporting their ability to age in place and enhancing their quality of life throughout the dying process (Gitlin et al. 2010; ElMokhallalati et al. 2023).

The place of death is widely recognized as a pivotal indicator of the quality of end-of-life care (Kinoshita et al. 2015; Health at Glance OECD, 2021; ElMokhallalati et al. 2023). For many, home is the preferred place of death, aligning with the values of person-centered care and autonomy, a preference that is particularly strong in East Asia (Ohmachi et al. 2015; Lee et al. 2018; Kao et al. 2022). Conversely, hospital admissions at the end of life are often burdensome; dementia is associated with a high risk of behavioral and psychiatric symptoms, delirium, and prolonged lengths of stay during hospitalization (Mukadam and Sampson 2011; Collier 2012; Sampson et al. 2014). Despite the risks associated with hospital deaths, they account for over 70% of deaths in Japan and Korea. Similarly, in Taiwan, PWD experience a significantly higher proportion (31%) of burdensome hospital transitions at the end of life compared to counterparts in the USA (19%) and the UK (16%) (Gozalo et al. 2011; Leniz et al. 2019; Chen et al. 2020).

In the context of traditional Chinese culture, end-of-life trajectories are further complicated by the belief that dying at home ensures spiritual blessings and prevents the soul from becoming lost (Lin et al. 2017). This deeply rooted cultural value drives a unique phenomenon known as “impending death discharge,” where terminally ill patients are discharged from the hospital specifically to “take their last breath at home” (Lin et al. 2017). This practice creates a challenge for healthcare research: patients recorded as home deaths in administrative data may have actually received aggressive hospital care until their final hours. Therefore, accurately evaluating the quality of end-of-life care in this context requires a nuanced understanding of these specific cultural dynamics.

While previous studies have examined factors influencing the place of death for PWD in Western cultures (Houttekier et al. 2010; Reyniers et al. 2015; Chen et al. 2022), little is known about the effect of HHC on home death in Asia, particularly when accounting for local cultural practices. This study aims to investigate the impact of a nationwide HHC program, its specific service characteristics, and its recent policy reform on the place of death among PWD in Taiwan. We hypothesized that HHC is associated with an increased likelihood of home death, and that this association is modulated by policy enhancements and cultural factors.

## Method

### Settings

National Health Insurance (NHI) in Taiwan is a universal healthcare scheme that has extended coverage to 99.9% of the population in the country since 1995. Primary HHC in Taiwan was also reimbursed by the NHI at the same year, providing clinical services including physician visits and nursing care for homebound people in the community (Li and Chang 2004). The criteria for individuals receiving primary HHC include: (1) possessing a restricted capacity for self-care, such as requiring assistance with over half of their activities of daily living; (2) having definite medical or nursing care needs, such as interventional tubes and ventilators care; and (3) dealing with chronic conditions that require extended nursing care after being discharged.

In 2016, the Taiwan government undertook a reform of home-based care services and introduced a new initiative titled the “Integrated Home-Based Medical Care (iHBMC)” program. This program was designed to more effectively address the diverse care needs of individuals who are disabled (Barthel Index score less than 60/100) or lived with an illness that leads to difficulties in seeking medical services outside of home. The iHBMC program is structured into three stages of service delivery: the first stage involves the introduction of a new category, Home-Based Primary Care (HBPC), which provided medical care via ordinary health visit for homebound people without specialized nursing care need; the second stage, known as HBPC Plus, which applies the same eligibility criteria with the old program, primary HHC, and presented enhanced services, targets patients with more complex care needs than those in HBPC; and the third stage offers an upgraded form of Home-based Palliative Care.

The iHBMC program enhances flexibility in drug prescriptions and care integration. It provides additional payments for case management and palliative care family meetings, benefiting both care recipients and caregivers. The program also monitors healthcare outcomes to improve care quality. However, people living in long-term care facilities were only eligible for receiving primary HHC and were excluded from the iHBMC program.

### Data source and ethics

The present study is one of the workstreams of the Home-based Longitudinal Investigation of the Multidisciplinary Team Integrated Care (HOLISTIC) study and is described in detail elsewhere (Liao et al. 2020). The National Health Insurance Research Database (NHIRD), which is derived from claims data of all NHI-reimbursed services, contains comprehensive healthcare data, including demographic details, diagnosed illnesses, and comprehensive records of both inpatient and outpatient healthcare services. The database employs the International Classification of Diseases, 9th Revision, Clinical Modification (ICD-9-CM) for diagnoses and procedures until 2016, after which ICD-10-CM codes were implemented (NHIA Statistical Report 2021).

### Study design and participants

**Figure 1.**
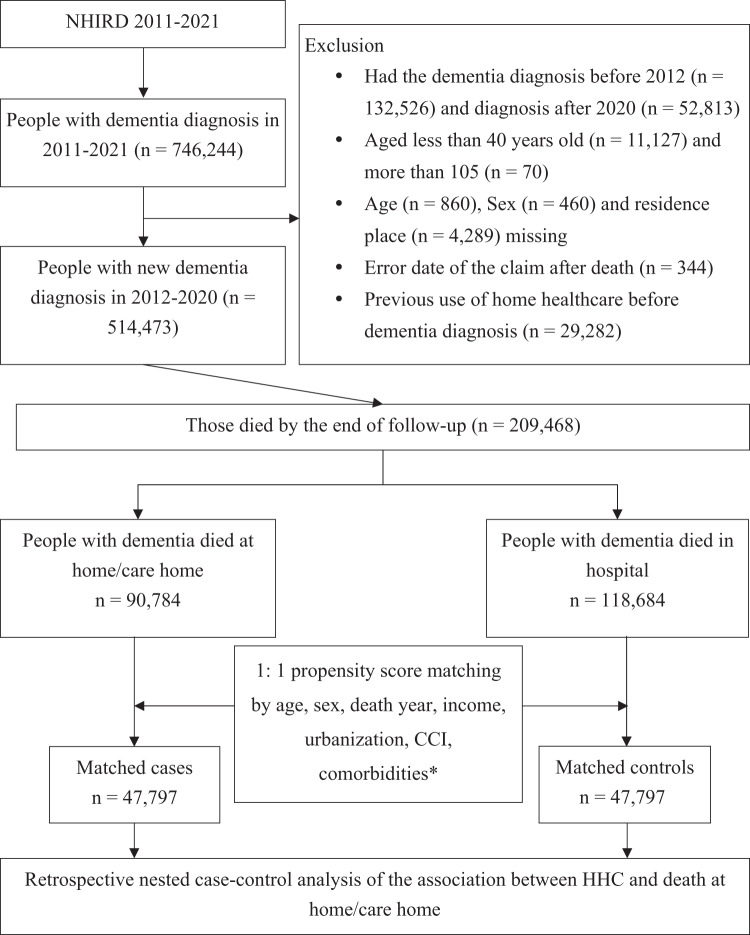
Schematic illustration of the source population selection, study design and cohort identification in PWD who had home death and hospital death. Figure 1 long description.

We conducted a nationwide cohort study using NHIRD over the period from January 1, 2011 to December 31, 2021. Individuals aged 40 years and older who had a new diagnosis of dementia were identified during this timeframe, based on ICD-9-CM and ICD-10-CM codes (Table A1 in Appendix A). The diagnosis was confirmed by the presence of at least 1 inpatient record or a minimum of 3 outpatient records with dementia codes within a 1-year period. Individuals were excluded if they had a diagnosis of dementia earlier than 2011, were under 40 or over 105 years of age, or had received HHC before the cohort entry date. Those with data registration errors, such as records of HHC or hospital discharge after death, or missing demographic information, were also excluded from this study (Figure 1).

CCI = Charlson Comorbidity Index, HHC = home healthcare, NHIRD = National Health Insurance Research Database.*Comorbidities included cancer, chronic obstructive pulmonary disease, liver cirrhosis/chronic liver disease, renal failure, cerebrovascular disease, coronary artery disease, atrial fibrillation, hypertension, diabetes, lower urinary tract syndrome, pressure sores, and malnutrition.

Next, PWD who died by the end of 2022 were further selected as the study candidates, and the index date was designated as the individual’s date of death. In addition, we performed a nested case–control analysis within the dementia cohort decedents to minimize the bias of identifying end-of-life outcomes.

### Exposure measurements and covariates

We reviewed all records of HHC received by PWD during the follow-up period before death. Characteristics of HHC, including program type; resource utilization groups (RUGs); total counts of services or services provided by different professionals; and duration and frequency of HHC, were presented and used in the subgroup analyses. The RUGs, categorized into 4 levels, were assessed based on records from the initial HHC visit, with care needs progressively increasing from level 1 to level 4 (Table A2) (The project of integrated home-based medical care, NHIA 2023).

Covariates included age, sex, comorbidities, the Charlson Comorbidity Index (Charlson et al. 1994), recent acute illnesses, interventional tube use, and healthcare utilization in the death year, and socioeconomic status (income and urbanization level of residence) at cohort entry. Comorbidities were identified based on the presence of at least 1 inpatient record or 3 outpatient records within 1 year prior to the index date and were classified using ICD-9-CM and ICD-10-CM codes. Recent acute illnesses, including pneumonia, urinary tract infection, and hip fracture, were identified within the 3 months preceding the index date. Use of interventional tubes indicates procedures that happened within 6 months before the index date. Healthcare utilization, including admissions and use of home-based palliative care, was identified between the cohort entry date and the index date. Income levels were determined by insurance premium categories, and urbanization level was defined according to population density and medical resources (Liu et al. 2006).

### Outcome measurements

Deaths occurring in hospitals were identified through data from the NHIRD, whereas deaths outside of hospitals, including in the home or care homes, were identified using Taiwan’s national cause-of-death database. A sensitivity analysis was performed to account for any outpatient records recorded 3 or more days prior to death.

The place of death for PWD was categorized into 2 groups – “home death” and “hospital death.” We explored 2 definitions of classifying home death and hospital death. Fundamentally, the “home death” group included individuals who passed away either at home or in a care home, while the “hospital death” group comprised those who died in a hospital setting.

Furthermore, cultural issue plays a distinctive role in shaping discussions about the place of death. To consider the Taiwan’s culture-specific phenomenon “impending death discharge,” where dying individuals are commonly discharged within 1–3 days before death to take their last breath at home (Tang et al. 2009). We further conducted a sensitivity analysis with reclassified definitions of home or hospital death. Individuals who were recorded as home deaths in the death certification, recorded as dying at home, but who had a hospital discharge within 3 days before death, were reclassified into the “hospital death” group.

### Statistical analysis

We used propensity score matching at a 1:1 ratio to balance characteristics between groups of home and hospital death. A caliper width of 0.2 standard deviations of the logit of the propensity score was used for matching (Austin 2011).

We further used conditional logistic regression to estimate the association between HHC and home death after adjusting for confounders and presented the adjusted odds ratio (aOR). Moreover, stratified analyses for potential factors of interest were estimated and demonstrated as forest plots. A 2-tailed *p* < 0.05 was considered statistically significant. The statistical analyses were performed using SAS software, version 9.4 (SAS Institute, Inc., Cary, NC, USA).

## Result

Table 1 presents the characteristics of the study cohort composed of PWD who died at home/care home or at a hospital before and after matching. Prior to matching, substantial differences were observed between groups; after 1:1 propensity score matching, demographic and health status characteristics were well balanced. Compared with the hospital death group, the home death group had lower frequency of emergency department visits (93.4% vs. 94.5%), lower rate of intensive care unit admission (45.9% vs. 58.9%), less endotracheal intubation (6.6% vs. 12.8%), and less tracheostomy application (0.8% vs. 1.9%) within 6 months before death.

**Table 1.** The characteristics of the included PWD who died at home or in the hospital Table 1 long description.

	Before matching (*n* = 209,468)					After matching (*n* = 95,594)		
	Home death *n* = 90,784		Hospital death *n* = 118,684		*p*	Home death *n* = 47,797	Hospital death *n* = 47,797	*p*
*Characteristics at time of death year*								
*Predisposing factors*								
Age, *n* (%)					<0.0001			1.0000
<75		14,620 (16.1)		23,591 (19.9)		7753 (16.2)	7753 (16.2)	
75–84		43,366 (47.8)		54,244 (45.7)		23,638 (49.5)	23,638 (49.5)	
≥85		32,798 (36.1)		40,849 (34.4)		16,406 (34.3)	16,406 (34.3)	
Sex (female), *n* (%)		47,799 (52.7)		54,610 (46.0)	<0.0001	24,767 (51.8)	24,767 (51.8)	1.0000
*Enabling factors*								
Income (TWD), *n* (%)					<0.0001			1.0000
Dependent		31,934 (35.2)		44,000 (37.1)		19,253 (40.3)	19,253 (40.3)	
< 20,000		16,920 (18.6)		39,780 (33.5)		11,242 (23.5)	11,242 (23.5)	
20,001–40,000		40,825 (45.0)		33,003 (27.8)		16,977 (35.5)	16,977 (35.5)	
≥40,001		1105 (1.2)		1901 (1.6)		325 (0.7)	325 (0.7)	
Urbanization, *n* (%)					<0.0001			1.0000
Urban		36,549 (40.3)		58,469 (49.3)		22,366 (46.8)	22,366 (46.8)	
Sub-urban		41,877 (46.1)		47,691 (40.2)		21,441 (44.9)	21,441 (44.9)	
Sub-rural		10,326 (11.4)		10,661 (9.0)		3645 (7.6)	3645 (7.6)	
Rural		2032 (2.2)		1863 (1.6)		345 (0.7)	345 (0.7)	
*Need factors*								
CCI, *n* (%)					<0.0001			1.0000
1–2		47,946 (52.8)		50,902 (42.9)		30,685 (64.2)	30,685 (64.2)	
3		16,949 (18.7)		25,143 (21.2)		7618 (15.9)	7618 (15.9)	
≥4		25,889 (28.5)		42,639 (35.9)		9494 (19.9)	9494 (19.9)	
LUTS, *n* (%)		8372 (9.2)		11,376 (9.6)	0.0048	2077 (4.3)	2077 (4.3)	1.0000
Pressure injuries, *n* (%)		8410 (9.3)		10,855 (9.1)	0.3560	2244 (4.7)	2244 (4.7)	1.0000
Malnutrition, *n* (%)		1096 (1.2)		1632 (1.4)	0.0008	75 (0.2)	75 (0.2)	1.0000
Recent acute events, *n* (%)								
Pneumonia						10,687 (22.4)	20,186 (42.2)	<0.0001
UTI						6825 (14.3)	11,529 (24.1)	<0.0001
Hip fracture						556 (1.2)	633 (1.3)	0.0246

TWD = Taiwan Dollar, CCI = Charlson Comorbidity Index, LUTS = lower urinary tract syndrome, UTI = urinary tract infection.

The follow-up characteristics of PWD in 2 groups after matching, including HHC and hospital care utilization, are presented in Table 2. A notable shift was observed: the home-death group had a higher proportion of HBPC Plus and post-2016 primary HHC use (17.2% and 19.1%, respectively) than the hospital-death group.

**Table 2.** Follow-up characteristics among PWD in home or hospital death groups Table 2 long description.

	**Home death** *n* = 47,797	**Hospital death** *n* = 47,797	*p*
**Hospitalization within 7 days before death**, *n* (%)	18,015 (37.7)	34,039 (71.2)	<0.0001
= 0 (Discharged on death day)	13,451 (74.7)	30,767 (90.4)	
1–3	2646 (14.7)	1871 (5.5)	
4–7	1918 (10.7)	1401 (4.1)	
***HHC-related factors***			
**HHC use** (yes), *n* (%)	13,252 (27.7)	14,361 (30.1)	<0.0001
**Type of HHC**, *n* (%)			
Primary HHC (before 2016)	4053 (30.6)	4423 (30.8)	
Primary HHC (after 2016)	2537 (19.1)	1733 (12.1)	
Primary HHC (care home)	4386 (33.1)	6805 (47.4)	
HBPC plus	2276 (17.2)	1400 (9.8)	
**HHC count** (total counts/person), median (Q1–Q3)	9 (3–21)	9 (3–22)	0.1073
**Duration of HHC** (days), median (Q1–Q3)	267 (60–693)	288 (63–728)	0.0138
**Frequency of HHC** (counts /month/person), *n* (%)			<0.0001
≤1	3253 (24.5)	3940 (27.4)	
1–1.5	6181 (46.6)	6654 (46.3)	
1.5–2	1031 (7.8)	905 (6.3)	
>2	1086 (8.2)	987 (6.9)	
Only 1 count	1701 (12.8)	1875 (13.1)	
**RUG of first HHC**, *n* (%)			<0.0001
RUG 1	938 (7.1)	656 (4.6)	
RUG 2	9877 (74.5)	11,033 (76.8)	
RUG 3	2358 (17.8)	2604 (18.1)	
RUG 4	79 (0.6)	68 (0.5)	
**Level of HHC agency**, *n* (%)			<0.0001
Community home care institution	7654 (57.8)	9193 (64.0)	
Hospital	5512 (41.6)	5062 (35.2)	
Missing	86 (0.6)	106 (0.7)	

HHC = home healthcare, HBPC = Home-Based Primary Care, Q1 = first quartile, Q3 = third quartile, RUG = resource utilization group.

Table 3 demonstrates the regression analysis examining the association between HHC and home death (results of the univariate model shown in the appendix Table A3). Without considering cultural factors, HHC use was associated with higher odds of home death (aOR = 1.17; 95% CI: 1.13–1.21). When impending death discharge was taken into account, the association strengthened substantially (aOR = 1.67; 95% CI: 1.59–1.75).

**Table 3.** Conditional logistic regression analysis for the odds of home death in PWD who received HHC compared to those without HHC (reference group) Table 3 long description.

	**Home death** (WITHOUT cultural consideration)		**Home death** (WITH cultural consideration)	
	aOR	(95% CI)	aOR	(95% CI)
HHC	1.17	(1.13–1.21)	1.67	(1.59–1.75)
Recent acute events				
Pneumonia	0.52	(0.50–0.54)	0.34	(0.32–0.36)
UTI	0.81	(0.78–0.84)	0.75	(0.70–0.80)
Hip fracture	0.98	(0.86–1.12)	1.01	(0.84–1.21)
Duration of dementia to death (years)				
0–0.5	[Reference]		[Reference]	
0.5–2	0.90	(0.86–0.94)	0.88	(0.81–0.94)
2–3.5	0.94	(0.89–0.98)	0.88	(0.81–0.95)
>3.5	0.99	(0.94–1.05)	0.92	(0.85–0.99)
Medical events before death				
Any previous hospitalization	0.77	(0.72–0.83)	N/A^a^	
Any ICU admission	0.84	(0.81–0.86)	0.72	(0.69–0.75)
Any home palliative care use	2.38	(2.21–2.57)	3.89	(3.51–4.32)
Enteral tube insertion or Tube feeding	0.51	(0.49–0.53)	0.33	(0.31–0.34)
Urinary catheterization	0.82	(0.79–0.85)	0.67	(0.64–0.71)
Endotracheal intubation or Tracheostomy	0.84	(0.80–0.88)	0.77	(0.71–0.82)

CI = confidence interval, aOR = adjusted odds ratio, HHC = Home healthcare, UTI = urinary tract infection, ICU = Intensive care unit.

^a^N/A: all patients in the “Home death” group had previous hospitalization before death, leading to a meaningful OR value that could not be calculated.

**Figure 2.**
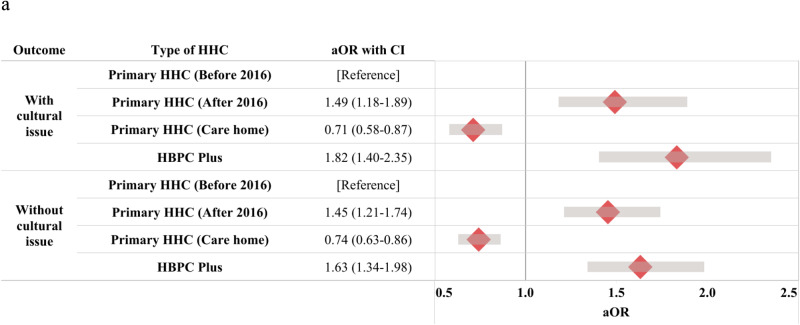
Forrest plot of odds of home death among PWD receiving different HHC programs before and after 2016 in Taiwan. Figure 2 long description.

As shown in Figure 2, compared with pre-2016 primary HHC, both HBPC Plus and post-2016 primary HHC were associated with a greater likelihood of home death (HBPC Plus: aOR = 1.63; 95% CI: 1.34–1.98; post-2016 primary HHC: aOR = 1.45; 95% CI: 1.21–1.74). This pattern persisted and became stronger when cultural reclassification was applied (aOR = 1.82 and 1.49, respectively).

Appendix Table A4 further presents subgroup analyses. More frequent HHC visits were associated with a trend toward higher odds of home death, and HHC delivered by hospital-based teams showed a higher likelihood of home death compared with community-based teams.

## Discussion

To the best of our knowledge, this is the first nationwide cohort study in Asia to examine how HHC and its policy reform influence the place of death among PWD, while explicitly integrating culturally rooted end-of-life practices. Three key findings emerged. First, HHC use was associated with a higher likelihood of home death. Second, the 2016 iHBMC reform – particularly HBPC Plus – further strengthened this association. Third, when impending death discharge was incorporated into outcome classification, the association became even more pronounced, underscoring the need to interpret place-of-death data within Taiwan’s cultural context.

### The role of HHC and its reform in home death

Consistent with findings from systematic reviews (Chen et al. 2022), our results demonstrate that HHC – despite not being designed as an end-of-life model – plays a meaningful role in enabling home death among PWD. Continuous medical support, timely clinical assessments, and the availability of urgent visits likely reduce unnecessary hospital transfers and help families manage terminal deterioration at home (Ritchie and Leff 2018; Chen et al. 2020). The strengthened association observed after 2016 suggests that the iHBMC reforms enhanced the capability of HHC teams to support end-of-life care (The project of integrated home-based medical care, NHIA 2023). Expanded eligibility, flexible visit frequencies, greater physician involvement from hospitals and community clinics, and improved interdisciplinary coordination may have allowed teams to anticipate complications earlier and stabilize patients at home more effectively (Liao et al. 2020).

Moreover, the availability of family-centered practices, including advanced care planning and hospice consultations, was reimbursed in the reformed HHC program (The project of integrated home-based medical care, NHIA 2023). Medical care teams may have incentives to initiate family meetings for future care planning for HPBC Plus recipients. Along with the legislation and mass communication of the Patient Right to Autonomy Act in 2016, patients, family members, and clinicians may be encouraged to have conversations of end-of-life care in both HBPC Plus and primary HHC (Cheng et al. 2020). These elements enabled patients and caregivers to gain a clearer understanding of the life trajectory and align medical decisions with personal and cultural values (Cheng et al. 2018). This, in turn, may increase the likelihood of receiving home hospice care and contribute to a greater tendency to die at home (Jennings et al. 2019).

Our finding that hospital-based HHC teams were associated with higher odds of home death aligns with prior research showing that medically intensive teams enable smoother transitions across care settings (Song 2022). Greater access to opioids and psychotropic medications, more end-of-life expertise, better interdisciplinary support, and the availability of 24-hour coverage may contribute to this effect (Gomes et al. 2013; Møller et al. 2018; Chen et al. 2020). Similarly, higher visit frequency likely reflects active symptom monitoring and more reliable clinical support, reducing late transitions to hospital (Cheng et al. 2010; Chen et al. 2020).

### *Cultural dynamics and home death in* *Taiwan*

Cultural beliefs strongly shaped end-of-life patterns in this study. In Taiwan, impending death discharge – returning home to die within 1–3 days – is a deeply rooted practice well-documented in terminally ill patients (Lin et al. 2017; Hsiao et al. 2022). Incorporating this cultural phenomenon into outcome definitions meaningfully altered effect estimates in our study. Our study suggests that impending death discharge should be considered as a key factor when investigating the impact of healthcare delivery on the place of end-of-life care and death.

For PWD, late burdensome transition, which was defined as any hospitalization or emergency room visits in the last 3 days of life, is a poor-quality indicator of end-of-life care and may lead to the risk of impending death discharge (Gozalo et al. 2011). In this study, HHC was associated with a stronger odds of home death when “impending death discharge” issue is considered than when it is not, indicating the HHC might reduce late burdensome hospital transition in the last 3 days of life and improve continuity of end-of-life care at home (Chen et al. 2020).

### Strength and limitations

Major strengths include the large, nationwide sample; comprehensive claims data; and the use of propensity score matching to enhance comparability between groups. The study also introduces a culturally informed analytic approach to place-of-death research, which has rarely been addressed in dementia populations.

Limitations include the lack of clinical data on dementia severity, functional status, and social support; the inability to distinguish dementia subtypes; and the absence of information on the preferred place of death. Generalizability to countries with different HHC systems or cultural practices may be limited.

## Conclusion

HHC substantially increases the likelihood of home death among PWD in Taiwan, especially following the implementation of the iHBMC reform. Key components – including flexible visit frequencies, integration with hospital-based physicians, and family-centered planning – appear important for supporting end-of-life care at home. Future mixed-methods research is needed to examine how HHC shapes the lived end-of-life experience and concordance between preferred and actual place of care and death.

## Supporting information

10.1017/S1478951526102491.sm001 Chen et al. supplementary material Chen et al. supplementary material

## Supplementary material

The supplementary material for this article can be found at https://doi.org/10.1017/S1478951526102491.

## Appendix

### Appendix

**Table A1.** List of International Classification of Diseases, Ninth Revision, Clinical Modification (ICD-9-CM) code and the 10th Revision (ICD-10-CM) code of the diseases Table A1 long description.

Disease	ICD − 9-CM code	ICD − 10-CM code
Dementia	290.0, 290.1, 290.2, 290.3, 290.4, 294.0, 294.1, 294.8, 331.0, 331.1, 331.2, 331.7, 331.82, 797	F01, F02, F03, F04, F06.1, F06.8, G30, G31.0, G31.1, G31.2, G31.8
Cancer	140 − 208	C00-C96, Z51, C7A, C7B, D03
Heart Failure	428, A289	I50、I110
Chronic obstructive pulmonary disease	490 − 496, 500 − 505, 506.4	J40-J47, J60-J67
Liver cirrhosis/ chronic liver disease	572.2 − 572.8, 456.0 − 456.2	K72.1, K72.9, K76.6, K76.7 K76.8, I85.01, I85.00, I85.10, I85.11
Renal failure	582 − 583, 585 − 586, 588	N03, N05, N06, N07, N16, N19, N25
Cerebrovascular disease (Stroke)	430 − 438	G45-G46, I60-I69
Coronary artery disease	410 − 414	I20-I25
Atrial fibrillation	427.31, 427.32	I48
Hypertension	401 − 405	I10-I13, I15-I16
Diabetes	250, 251	E08–E14
Lower Urinary Tract Symptoms	596.51, 625.6, 788.32, 788.31, 788.4, 788.36, 788.43, 788.33, 788.2, 788.6, 788.35	N32.81, N39.3, N39.41, R35.0, R35.8, N39.44, R35.1, N39.46, R33.0, R33.8, R33.9, R39.11, R39.12, R39.13, R39.15, R39.16, R39.19, N39.43
Pressure sores	707	L89
Malnutrition	260 − 262, 263.x	E40-E43, E44.x, E45, E46, E64
Pneumonia	466, 480 − 486, 507.0	J12-J18, J20-J22, J69.0
Urinary tract infection	590, 595 (exclude 595.1, 595.2, 595.82), 597, 599.0	N10, N30 (exclude N30.1, N30.2, N30.4), N34, N39.0
Hip fracture	820.21, 820.31, 820.8, 820.9, 821.00, 821.01, 821.10	S72.0, S72.1, S72.2, S72.3, S72.7, S72.8, S72.9, S73.0

### Appendix

**Table A2:** Specialized care services and Resource utilization group Table A2: long description.

Specialized care services	Resource utilization group (RUG)
Tracheostomy care	RUG 1: require ordinary healthcare visits only
Urinal indwelling catheterization and/or catheter change	RUG 2: require 1 specialized care service
Nasogastric tube insertion and/or change	RUG 3: require 2 specialized care services
Intravenous fluid supply	RUG 4: require 3 or more specialized care services
Bladder irrigation	
Wound care or debridement for stage 3 or 4 pressure injuries	
Colostomy, ileostomy, or urostomy care (e.g., an ileal conduit)	
Post-colon resection care	

### Appendix

**Table A3.** Conditional logistic regression analysis for the chance/odds of home death in people with dementia who received home healthcare compared with those who did not receive home healthcare services (reference group) in the univariate model Table A3 long description.

	Univariate model (WITHOUT cultural consideration)		Univariate model (WITH cultural consideration)	
	OR	(95% CI)	OR	(95% CI)
HHC	0.89	(0.86 − 0.91)	1.05	(1.01 − 1.08)
Recent acute events				
Pneumonia	0.32	(0.31 − 0.33)	0.17	(0.16 − 0.18)
UTI	0.48	(0.47 − 0.50)	0.34	(0.32 − 0.35)
Hip fracture	0.88	(0.78 − 0.98)	0.84	(0.73 − 0.97)
Duration of dementia to death (years)				
0 − 0.5	[Reference]		[Reference]	
0.5 − 2	0.98	(0.94 − 1.02)	1.05	(0.99 − 1.11)
2 − 3.5	1.00	(0.95 − 1.05)	1.06	(1 − 1.13)
>3.5	1.06	(1.01 − 1.11)	1.13	(1.07 − 1.2)
Medical events before death				
Any previous hospitalization	0.41	(0.38 − 0.44)	NA	
Any ICU admission	0.57	(0.56 − 0.59)	0.50	(0.49 − 0.52)
Any home palliative care use	2.54	(2.37 − 2.73)	3.62	(3.32 − 3.94)
Enteral tube insertion or Tube feeding	0.31	(0.30 − 0.32)	0.18	(0.17 − 0.19)
Urinary catheterization	0.44	(0.43 − 0.46)	0.28	(0.27 − 0.29)
Endotracheal intubation or Tracheostomy	0.47	(0.45 − 0.49)	0.24	(0.22 − 0.26)

**Note**. CI = confidence interval, OR = odds ratio, HHC = Home healthcare, UTI = urinary tract infection, ICU = Intensive care unit.

### Appendix

**Table A4.** Odds of home death in people with dementia who received home healthcare stratified by the characteristics of home healthcare Table A4 long description.

	Home death (WITHOUT cultural consideration)		Home death (WITH cultural consideration)	
	aOR	(95% CI)	aOR	(95% CI)
Frequency of HHC visit (counts/month/person)				
≤1	[Reference]		[Reference]	
1 − 1.5	1.05	(0.94 − 1.17)	1.06	(0.92 − 1.23)
1.5 − 2	1.35	(1.11 − 1.64)	1.32	(1.02 − 1.70)
>2	1.35	(1.11 − 1.64)	1.28	(0.99 − 1.65)
Resource utilization group (RUG) of first HHC				
RUG 1	[Reference]		[Reference]	
RUG 2	0.77	(0.64 − 0.92)	0.81	(0.65 − 1.01)
RUG 3	0.83	(0.68 − 1.01)	0.99	(0.77 − 1.27)
RUG 4	0.90	(0.47 − 1.72)	1.26	(0.57 − 2.80)
Level of HHC agency				
Community home care institution	[Reference]		[Reference]	
Hospital	1.36	(1.24 − 1.49)	1.22	(1.08 − 1.38)

**Notes**: CI = confidence interval, aOR = adjusted odds ratio, HHC = Home healthcare, HBPC = Home-Based Primary Care, RUG = Resource utilization group.

## Figure 1 Long description

The flowchart details the selection process for a study cohort from the NHIRD database between 2011 and 2021. It begins with 'People with dementia diagnosis in 2011-2021 (n = 746,244).' From this group, 'People with new dementia diagnosis in 2012-2020 (n = 514,473)' are identified. Exclusions are made for those diagnosed before 2012 (n = 132,526) and after 2020 (n = 52,813), those aged less than 40 or more than 105 (n = 11,127 and n = 70, respectively) and those with missing age (n = 860), sex (n = 460), or residence place (n = 4,289). Additional exclusions include error dates of claims after death (n = 344) and previous use of home healthcare before dementia diagnosis (n = 29,282). The remaining group, 'Those died by the end of follow-up (n = 209,468),' is split into 'People with dementia died at home/care home (n = 90,784)' and 'People with dementia died in hospital (n = 118,684).' A 1:1 propensity score matching is conducted by age, sex, death year, income, urbanization, CCI and comorbidities, resulting in 'Matched cases n = 47,797' and 'Matched controls n = 47,797.' The study is a retrospective nested case-control analysis of the association between HHC and death at home/care home.

Navigate back to Figure 1.

## Figure 2 Long description

Outcome With cultural issue Type of HHC Primary HHC (Before 2016) [Reference] Primary HHC (After 2016) 1.49 (1.18-1.89) Primary HHC (Care home) 0.71 (0.58-0.87) HBPC Plus 1.82 (1.40-2.35) Without cultural issue Type of HHC Primary HHC (Before 2016) [Reference] Primary HHC (After 2016) 1.45 (1.21-1.74) Primary HHC (Care home) 0.74 (0.63-0.86) HBPC Plus 1.63 (1.34-1.98) The x-axis is labeled aOR, with tick labels 0.5, 1.0, 1.5, 2.0 and 2.5. A vertical reference line is drawn at 1.0. Each row shows a diamond marker with a horizontal confidence interval bar.

Navigate back to Figure 2.

## Table 1 Long description

The table compares characteristics of people with dementia who died at home versus in hospital, shown before matching (90,784 home; 118,684 hospital) and after matching (47,797 in each group). Before matching, groups differed significantly across age, sex, income, urbanization, and comorbidity: hospital deaths had fewer females (46.0% vs 52.7%), more urban residents (49.3% vs 40.3%), and higher comorbidity (CCI ≥4: 35.9% vs 28.5%). Before matching, hospital deaths also had slightly higher rates of LUTS (9.6% vs 9.2%) and malnutrition (1.4% vs 1.2%), while pressure injuries were similar (9.1% vs 9.3%). After matching, distributions were exactly the same between settings for age, sex, income, urbanization, CCI, LUTS, pressure injuries, and malnutrition (all P=1.0000), indicating balance on these baseline factors. In the matched sample, recent acute events were more common among hospital deaths: pneumonia 42.2% vs 22.4%, UTI 24.1% vs 14.3%, and hip fracture 1.3% vs 1.2% (all statistically significant). P-values indicate statistical differences but do not convey effect size or causality, and the “recent acute events” section is only reported for the matched groups.

Navigate back to Table 1.

## Table 2 Long description

The table compares end-of-life care and home healthcare characteristics for two equal-sized dementia groups: home death (n=47,797) and hospital death (n=47,797), reporting counts/percentages and p-values. Hospitalization within 7 days before death was much higher for hospital deaths than home deaths (71.2% vs 37.7%; p<0.0001). Among those hospitalized, same-day discharge was more common in hospital deaths (90.4% vs 74.7%), while longer stays were more common in home deaths (1–3 days: 14.7% vs 5.5%; 4–7 days: 10.7% vs 4.1%). Home healthcare use was slightly higher in the hospital-death group (30.1% vs 27.7%; p<0.0001). Among home healthcare users, care-home primary home healthcare was more frequent in hospital deaths (47.4% vs 33.1%), while “after 2016” primary home healthcare (19.1% vs 12.1%) and HBPC plus (17.2% vs 9.8%) were more common in home deaths. Total home healthcare counts were similar (median 9 in both groups; p=0.1073), but duration was slightly longer for hospital deaths (median 288 vs 267 days; p=0.0138). Visit frequency distributions differed (p<0.0001), with hospital deaths having more ≤1 per month (27.4% vs 24.5%) and home deaths having more >2 per month (8.2% vs 6.9%). The first-visit resource utilization group differed (p<0.0001), with hospital deaths higher in RUG 2 (76.8% vs 74.5%) and lower in RUG 1 (4.6% vs 7.1%). Agency level also differed (p<0.0001): community institutions were more common for hospital deaths (64.0% vs 57.8%), while hospital-based agencies were more common for home deaths (41.6% vs 35.2%), with minimal missing data.

Navigate back to Table 2.

## Table 3 Long description

The table reports adjusted odds ratios (aORs) with 95% confidence intervals for home death among people with dementia, comparing those who received home healthcare with those who did not, under two models: without and with cultural consideration. Home healthcare is associated with higher odds of home death in both models (aOR 1.17, 95% CI 1.13–1.21; and 1.67, 1.59–1.75). Recent acute events generally reduce the odds of home death, especially pneumonia (0.52, 0.50–0.54; and 0.34, 0.32–0.36) and UTI (0.81, 0.78–0.84; and 0.75, 0.70–0.80), while hip fracture is near null (0.98, 0.86–1.12; and 1.01, 0.84–1.21). Using 0–0.5 years from dementia diagnosis to death as the reference, longer durations show slightly lower or near-null odds, with the >3.5 years category close to 1.0 without cultural consideration (0.99, 0.94–1.05) and modestly lower with it (0.92, 0.85–0.99). Prior hospitalization is associated with lower odds without cultural consideration (0.77, 0.72–0.83), but an odds ratio is not available in the culturally adjusted model. ICU admission is associated with lower odds (0.84, 0.81–0.86; and 0.72, 0.69–0.75). Home palliative care use shows the largest increase in odds of home death (2.38, 2.21–2.57; and 3.89, 3.51–4.32). Several intensive interventions are associated with lower odds of home death in both models, including enteral tube insertion/tube feeding (0.51 to 0.33), urinary catheterization (0.82 to 0.67), and intubation/tracheostomy (0.84 to 0.77).

Navigate back to Table 3.

## Table A1 Long description

This table maps diseases to their ICD-9-CM and ICD-10-CM codes. It includes conditions such as dementia, cancer, heart failure, chronic obstructive pulmonary disease, liver disease, renal failure, stroke, coronary artery disease, atrial fibrillation, hypertension, diabetes, urinary symptoms, pressure sores, malnutrition, pneumonia, urinary tract infection, and hip fracture. Codes are presented as ranges or individual values, providing a cross-reference between the two classification systems.

Navigate back to Table A1.

## Table A2: Long description

This table categorizes specialized care services into resource utilization groups. RUG 1 includes patients requiring only ordinary care. RUG 2 includes those requiring one specialized service such as tracheostomy care or catheterization. RUG 3 includes those requiring two services such as nasogastric tube care. RUG 4 includes patients requiring three or more services, including intravenous fluids, bladder irrigation, advanced wound care, ostomy care, and post-colon resection care. The table reflects increasing care complexity with higher RUG levels.

Navigate back to Table A2:.

## Table A3 Long description

This table presents univariate logistic regression results for the odds of home death among people with dementia receiving home healthcare. It compares models with and without cultural consideration. Variables include home healthcare use, recent acute events such as pneumonia, urinary tract infection, and hip fracture, duration of dementia, and medical events before death such as hospitalization, ICU admission, palliative care, and medical interventions. Odds ratios and confidence intervals are reported. Results show that infections and intensive interventions are associated with lower odds of home death, while palliative care is associated with higher odds. Cultural adjustment alters some associations.

Navigate back to Table A3.

## Table A4 Long description

This table presents adjusted odds ratios for home death among dementia patients receiving home healthcare, stratified by visit frequency, resource utilization group, and agency level. Results are shown with and without cultural consideration. Increased visit frequency is generally associated with higher odds of home death. Resource utilization groups show variable associations. Receiving care from hospitals compared to community home care institutions is associated with higher odds of home death. Confidence intervals indicate the precision of estimates.

Navigate back to Table A4.
