# Impact of home healthcare reform on place of death for people with dementia: A nationwide cohort study accounting for cultural factors of impending death discharge – CORRIGENDUM

**DOI:** 10.1017/S1478951526102909

**Published:** 2026-06-10

**Authors:** Chia-hung Chen, Elizabeth L. Sampson, Jung-Yu Liao, Chung-Han Ho, Wei-Zhe Tseng, Irene Petersen, Yi-Chi Wang, Yi-Chen Lai, Yu-Han Chen, Hung-Yi Chiou, Chao A. Hsiung, Sang-Ju Yu, Christine Ritchie, Ping-Jen Chen

DOI: https://doi.org/10.1017/S1478951526102491. Published by Cambridge University Press, 11 May 2026

The authors apologise for the omission of this funding information.

Financial Support:

This work was supported by the National Science and Technology Council of Taiwan (112-2410-H-037-005-MY3), Ministry of Health and Welfare of Taiwan (TSOHHC112HaH), and a grant from Kaohsiung Medical University Hospital (KMUH114-4R83). The funders had no role in study design, methods, data collection, analysis, or preparation of the manuscript.

The original article has been updated.
