# Supplementary material for: Impact of home healthcare reform on place of death for people with dementia: A nationwide cohort study accounting for cultural factors of impending death discharge
Source: Palliat Support Care. 2026 May 11;24:e139. doi: 10.1017/S1478951526102491 (PMC13202396; doi:10.1017/S1478951526102491)
Supplement: Chen et al. supplementary material [file S1478951526102491sup001.docx]

**Appendix**

**Table S1. Odds of home death in people with dementia who received home healthcare stratified by the characteristics of home healthcare**

|  | **Home death**  (WITHOUT cultural consideration) | | **Home death**  (WITH cultural consideration) | |
| --- | --- | --- | --- | --- |
|  | aOR | (95% CI) | aOR | (95% CI) |
| Frequency of HHC visit (counts/month/person) |  |  |  |  |
| <=1 | [Reference] | | [Reference] | |
| 1-1.5 | 1.05 | (0.94-1.17) | 1.06 | (0.92-1.23) |
| 1.5-2 | 1.35 | (1.11-1.64) | 1.32 | (1.02-1.70) |
| >2 | 1.35 | (1.11-1.64) | 1.28 | (0.99-1.65) |
| Resource utilization group (RUG)  of first HHC, n (%) |  |  |  |  |
| RUG 1 | [Reference] | | [Reference] | |
| RUG 2 | 0.77 | (0.64-0.92) | 0.81 | (0.65-1.01) |
| RUG 3 | 0.83 | (0.68-1.01) | 0.99 | (0.77-1.27) |
| RUG 4 | 0.90 | (0.47-1.72) | 1.26 | (0.57-2.80) |
| Level of HHC agency |  |  |  |  |
| Community home care institution | [Reference] | | [Reference] | |
| Hospital | 1.36 | (1.24-1.49) | 1.22 | (1.08-1.38) |

*Note.* CI = confidence interval, aOR = adjusted odds ratio, HHC = Home healthcare, HBPC = Home-Based Primary Care, RUG = Resource utilization group
